# 4‐Octyl Itaconate Restores Microglial Efferocytosis by Suppressing NRF2/PD‐L1‐Dependent Neutrophil NET Formation in Sepsis‐Associated Encephalopathy

**DOI:** 10.1002/cns.71268

**Published:** 2026-11-10

**Authors:** Na Meng, Xixue Zhang, Kaiwen Tian, Yue Zhou, Lei Gao, Fafa Sun, Yulin Zhang, Lina Huang, Weidong Gu

**Affiliations:** ^1^ Department of Anesthesiology Huadong Hospital, Fudan University Shanghai China; ^2^ Department of Anesthesiology Shanghai General Hospital, Shanghai Jiao Tong University Shanghai China

**Keywords:** 4‐octyl itaconate, microglial efferocytosis, neutrophil extracellular traps, sepsis‐associated encephalopathy

## Abstract

**Background:**

Sepsis‐associated encephalopathy (SAE) is a frequent neurological complication of sepsis characterized by acute neurobehavioral abnormalities and persistent cognitive impairment. Although 4‐octyl itaconate (4‐OI) possesses potent immunomodulatory properties, its neuroprotective effects and underlying mechanisms in SAE remain unclear.

**Methods:**

Sepsis was induced by cecal ligation and puncture (CLP), and mice were treated with 4‐OI. Behavioral tests were used to assess anxiety‐like behavior and cognitive function. Transcriptomic and single‐cell RNA‐seq analyses were performed to characterize efferocytosis‐related changes in the septic hippocampus. Western blot, immunofluorescence, flow cytometry, experiments using primary neutrophils and microglia, pharmacological interventions, and *Padi4* ^−/−^ mice were used to investigate NET formation and microglial efferocytosis.

**Results:**

4‐OI improved neurobehavioral and cognitive outcomes, reduced hippocampal injury, blood–brain barrier disruption, and neuroinflammation, and suppressed NET accumulation in the septic hippocampus and LPS‐stimulated neutrophils. NRF2 inhibition weakened the effects of 4‐OI on neutrophil PD‐L1 expression and NET formation, whereas PD‐L1 neutralization reduced NET formation. Transcriptomic analyses identified microglia/myeloid‐associated efferocytosis programs. NETs impaired microglial efferocytosis, while DNase I treatment or PAD4 deficiency preserved apoptotic cell clearance.

**Conclusions:**

4‐OI protects against septic brain injury and cognitive dysfunction by activating NRF2, suppressing PD‐L1‐mediated NET formation, and mitigating NET‐associated impairment of microglial efferocytosis.

## Graphical abstract

Sepsis‐associated encephalopathy is driven in part by neutrophil PD‐L1–dependent NET accumulation, which impairs microglial efferocytosis and promotes neuroinflammation. 4‐Octyl itaconate activates NRF2, suppresses neutrophil PD‐L1 and NET formation, restores apoptotic‐cell clearance, and alleviates cognitive dysfunction, identifying the NRF2/PD‐L1/NET axis as a potential therapeutic target.

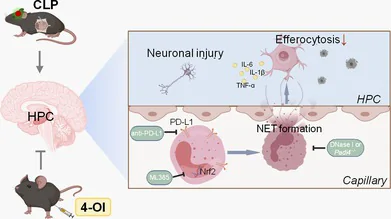

## 1 Introduction

Sepsis‐associated encephalopathy (SAE) is a frequent neurological complication of sepsis, characterized by acute disturbances of consciousness and long‐term cognitive impairment [1, 2]. Despite its clinical importance, SAE lacks specific therapeutic interventions and its pathogenesis remains incompletely understood.

Dysregulated neuroinflammatory responses are increasingly recognized as major contributors to the pathogenesis of SAE. As resident immune cells of the central nervous system, microglia contribute to tissue homeostasis through efferocytosis, a specialized process that clears apoptotic cells before loss of membrane integrity [3, 4]. Impaired microglial efferocytosis may promote apoptotic cell accumulation, secondary necrosis, and persistent neuroinflammation [5, 6], suggesting that preservation of microglial efferocytic function may help limit sepsis‐related neurological injury.

Neutrophil extracellular traps (NETs) are web‐like chromatin structures released by activated neutrophils [7, 8]. Although NETs contribute to pathogen containment, their excessive or persistent accumulation promotes endothelial injury, immunothrombosis, and organ dysfunction [9, 10]. Elevated circulating NET levels have been documented in septic patients and mice subjected to CLP and are associated with disease severity and tissue injury [11, 12]. In experimental SAE, hippocampal neutrophil infiltration and NET formation contribute to blood–brain barrier disruption, microglial activation, neuronal apoptosis, and memory impairment [12]. Furthermore, NETs have been shown to activate microglial inflammatory signaling and impair clearance functions in other brain injury models [13], and defective NET clearance has been linked to impaired efferocytosis in atherosclerosis [14]. However, whether NETs directly impair microglial efferocytosis during sepsis remains unclear.

Itaconate is a metabolic intermediate of the tricarboxylic acid (TCA) cycle that is produced in large amounts by LPS‐activated macrophages [15, 16]. Its cell‐permeable derivative, 4‐octyl itaconate (4‐OI), activates NRF2 signaling and exerts anti‐inflammatory effects [17, 18]. Recent studies have shown that 4‐OI suppresses NET formation in neutrophils under inflammatory conditions [19, 20, 21, 22], while NRF2 activation limits ROS‐dependent NET release [23]. PD‐L1 has also been identified as a marker of neutrophil dysfunction in sepsis, with elevated expression associated with immune dysregulation and poor prognosis [24]. These findings prompted us to hypothesize that 4‐OI may suppress aberrant NET release through NRF2‐mediated downregulation of neutrophil PD‐L1, thereby preserving microglial efferocytosis and attenuating neuroinflammation in SAE.

To test this hypothesis, we investigated the protective effects and mechanisms of 4‐OI in CLP‐induced SAE. We found that 4‐OI reduced hippocampal NET accumulation through NRF2‐mediated suppression of neutrophil PD‐L1 expression and improved neurobehavioral outcomes. We further demonstrated that NETs impaired microglial efferocytosis, whereas DNase I treatment or PAD4 deficiency preserved apoptotic cell clearance. These findings delineate an NRF2/PD‐L1/NETs axis linking neutrophil activation to defective microglial efferocytosis and identify 4‐OI as a pharmacological regulator of this pathway in SAE.

## 2 Materials and Methods

### 2.1 Animals

Male C57BL/6N mice aged 6–8 weeks were purchased from Shanghai Yishang Biotechnology Co. *Padi4* ^−/−^ mice were obtained from Shanghai Model Biology (NM‐KO‐190334). Sepsis was induced by cecal ligation and puncture (CLP). Briefly, mice were anesthetized, the cecum was exposed, ligated approximately 15 mm from the tip, punctured once with a 20‐gauge needle, and gently extruded to release a small amount of feces. Sham mice underwent laparotomy without ligation or puncture. Survival was monitored for 72 h after CLP or sham surgery. Survival curves were generated using the Kaplan–Meier method and compared using the log‐rank (Mantel–Cox) test. Mice that died before behavioral testing were excluded from behavioral analyses. Samples were collected at the indicated time points for subsequent analyses.

### 2.2 In Vivo Treatment

4‐OI (Selleck, S5929) was administered intraperitoneally at 50 mg/kg once daily for 2 days before CLP, with an additional injection on the day of surgery. In a separate post‐treatment experiment, mice received an intraperitoneal injection of 4‐OI at 50 mg/kg or saline 2 h after CLP or sham surgery [25, 26]. ML385 (Selleck, S8790) was injected intraperitoneally at 30 mg/kg 3 h before surgery to inhibit NRF2 signaling. For PD‐L1 blockade, mice received an intraperitoneal injection of a PD‐L1‐neutralizing antibody (10 mg/kg; Selleck, A2115) 3 h before CLP.

### 2.3 Cell Culture and Treatments

BV2 cells were obtained from Wuhan Servicebio Biotechnology Co. Ltd. and cultured in DMEM/F‐12 medium supplemented with 5% fetal bovine serum at 37°C with 5% CO₂. Bone marrow‐derived neutrophils (BMDNs) were isolated from mouse femurs and tibias using a mouse bone marrow neutrophil isolation kit (Solarbio, P8550) according to the manufacturer's instructions. BMDNs were treated with LPS (75 μg/mL; Sigma), 4‐OI (125 μM), ML385 (10 μM), or PD‐L1‐neutralizing antibody (10 μg/mL), as indicated. BV2 cells were exposed to isolated NETs at a DNA concentration of 5 μg/mL for 18 h.

### 2.4 Nissl Staining, Evans Blue Assay, and Brain Water Content

Nissl staining was performed on 10‐μm brain sections using cresyl violet to evaluate hippocampal neuronal injury. Blood–brain barrier permeability was assessed by Evans blue extravasation. Mice were injected with 2% Evans blue, perfused with cold PBS, and brain tissues were collected for imaging and quantitative measurement at 620 nm after formamide extraction. Brain edema was evaluated using the wet/dry weight method and expressed as brain water content.

### 2.5 Behavioral Tests

Open field, novel object recognition, and Y‐maze tests were performed to assess anxiety‐like behavior, locomotor activity, recognition memory, and spatial working memory. In the open field test, mice were allowed to explore freely for 5 min, and center‐zone entries, velocity, and total distance were recorded. In the novel object recognition test, mice explored two identical objects for 5 min, followed by a 4‐h interval and replacement of one object with a novel object. The recognition index was calculated from novel‐object exploration. In the Y‐maze test, mice freely explored the three‐arm maze for 8 min, and spontaneous alternation was calculated as the percentage of consecutive entries into three different arms.

### 2.6 Immunofluorescence Staining

For tissue immunofluorescence, hippocampal samples were fixed in 4% paraformaldehyde, cryoprotected, embedded in OCT, and sectioned at 10 μm. For cell immunofluorescence, cultured cells were fixed with 4% paraformaldehyde and permeabilized with 0.1% Triton X‐100. Samples were blocked with 1% BSA, incubated with primary antibodies overnight at 4°C, followed by fluorescence‐conjugated secondary antibodies. Nuclei were counterstained with DAPI, and images were captured using fluorescence microscopy.

### 2.7 Western Blotting

Cells or hippocampal tissues were lysed in lysis buffer containing protease inhibitors. Equal amounts of protein were separated by SDS‐PAGE and transferred to PVDF membranes. Membranes were blocked with 5% non‐fat milk, incubated with primary antibodies overnight at 4°C, followed by HRP‐conjugated secondary antibodies. Protein bands were visualized using enhanced chemiluminescence. Antibody information is provided in Table S1.

### 2.8 RT‐qPCR

Total RNA was extracted using TRIzol reagent, and 1 μg RNA was reverse‐transcribed using the ReverTra Ace kit (TOYOBO). RT‐qPCR was performed on a LightCycler 480 system using Power SYBR Green Master Mix. Gene expression was normalized to *Gapdh* and calculated using the 2^−ΔCt^ method. Primer sequences are listed in Table S2.

### 2.9 RNA Sequencing and Bioinformatic Analysis

Total RNA was extracted from hippocampal tissues, and RNA‐seq libraries were constructed and sequenced on the Illumina NovaSeq 6000 platform. Clean reads were aligned to the mouse genome, and differentially expressed genes were identified using DESeq2. Public transcriptomic datasets were obtained from GEO. The GSE167610 hippocampal dataset and an in‐house CLP hippocampal RNA‐seq dataset were analyzed for cross‐cohort validation. The *p* values were adjusted for multiple testing using the Benjamini–Hochberg false discovery rate procedure. Genes with an adjusted *p* value/FDR < 0.05 and an absolute log_2_ fold change ≥ 1 were defined as differentially expressed genes. DEGs were then used for functional and pathway enrichment analyses using TopGO and the KEGG database. Enriched GO terms and KEGG pathways with *p* < 0.05 were considered statistically significant. For single‐cell analysis, GSE311977 was used to examine cell‐type‐specific enrichment of efferocytosis‐related transcriptional programs.

This hippocampal RNA‐sequencing dataset was previously generated and used in our published study [27], in which the raw sequencing data were provided as Supplementary File 2.

### 2.10 Primary Microglia Isolation and Culture

Primary microglia were isolated from neonatal C57BL/6N mice aged 1–3 days. Briefly, forebrains were dissected in ice‐cold PBS, and meninges and visible blood vessels were removed. The tissues were enzymatically dissociated with 0.25% trypsin–EDTA and filtered through a 70‐μm cell strainer. After centrifugation, cells were resuspended in DMEM/F‐12 supplemented with 10% fetal bovine serum and 1% penicillin–streptomycin and seeded into poly‐L‐lysine‐coated culture flasks to establish mixed glial cultures. After 10 days, microglia were collected from the mixed glial cultures by gentle shaking, centrifuged, and replated for subsequent efferocytosis assays.

### 2.11 Efferocytosis Assay

For in vitro efferocytosis assays, primary microglia or BV2 cells (5 × 10^5^ cells/well) were exposed to NETs (5 μg/mL NET‐DNA) for 18 h. Where indicated, NETs were pretreated with DNase I (100 U/mL, 30 min, 37°C). CFSE‐labeled apoptotic HT22 cells or BMDNs were then cocultured with primary microglia or BV2 cells, respectively, at a 1:5 ratio for 3 h. After washing, efferocytosis was assessed by fluorescence microscopy or flow cytometry. For flow cytometry, cells were Fc‐blocked, fixed, permeabilized, and stained for Iba‐1 followed by an ABflo 647‐conjugated secondary antibody. CFSE^+^Iba‐1^+^ microglia were quantified using a BD Accuri C6 Plus. For microscopy, images were acquired using a Leica THUNDER system, with at least three random fields per well analyzed.

### 2.12 Chromatin Immunoprecipitation (ChIP)‐qPCR

Chromatin immunoprecipitation was performed using the BeyoChIP Assay Kit (Beyotime, P2080S). Approximately 1 × 10^6^ cells were formaldehyde‐crosslinked and sonicated. Chromatin was incubated overnight at 4°C with the indicated antibody or normal IgG, followed by Protein A/G magnetic bead capture. After washing and elution, purified immunoprecipitated DNA was subjected to conventional PCR or qPCR using primers listed in Table S3.

### 2.13 Statistical Analysis

Statistical analyses were performed using GraphPad Prism 9 software (GraphPad Software, La Jolla, CA, USA). Data are presented as the mean ± SD unless otherwise indicated. For comparisons between two groups, an unpaired two‐tailed Student's *t*‐test was used. For multiple‐group comparisons without a factorial design, one‐way ANOVA followed by Tukey's multiple comparisons test was performed. For experiments with a 2 × 2 factorial design, two‐way ANOVA followed by Tukey's multiple comparisons test was used to evaluate the main effects of each factor and their interaction. Data normality and homogeneity of variance were assessed before statistical analysis. Exact sample sizes are indicated in the figure legends. Detailed statistical information, including the test used, *F* or *t* values, degrees of freedom, exact *p* values, and post hoc multiple‐comparison procedures, is provided in Supplementary File 1. A value of *p* < 0.05 was considered statistically significant.

## 3 Results

### 3.1 4‐OI Protects Against CLP‐Induced Neurological Injury

To determine whether 4‐OI protects against sepsis‐associated neurological dysfunction, mice were subjected to CLP followed by behavioral, histological, and blood–brain barrier assessments (Figure 1a). Kaplan–Meier survival analysis showed that CLP induced mortality within 72 h after surgery. 4‐OI‐treated mice showed a numerically higher survival proportion than CLP mice, but this difference was not statistically significant by the log‐rank test (Figure S1). In the open field test, CLP mice exhibited fewer center‐zone entries, reduced movement velocity, and shorter total distance traveled, indicating anxiety‐like behavior and impaired locomotor activity (Figure 1b–e). CLP also reduced the discrimination index in the novel object recognition test and spontaneous alternation percentage in the Y‐maze test, both of which were improved by 4‐OI pretreatment (Figure 1f,g).

**FIGURE 1:**
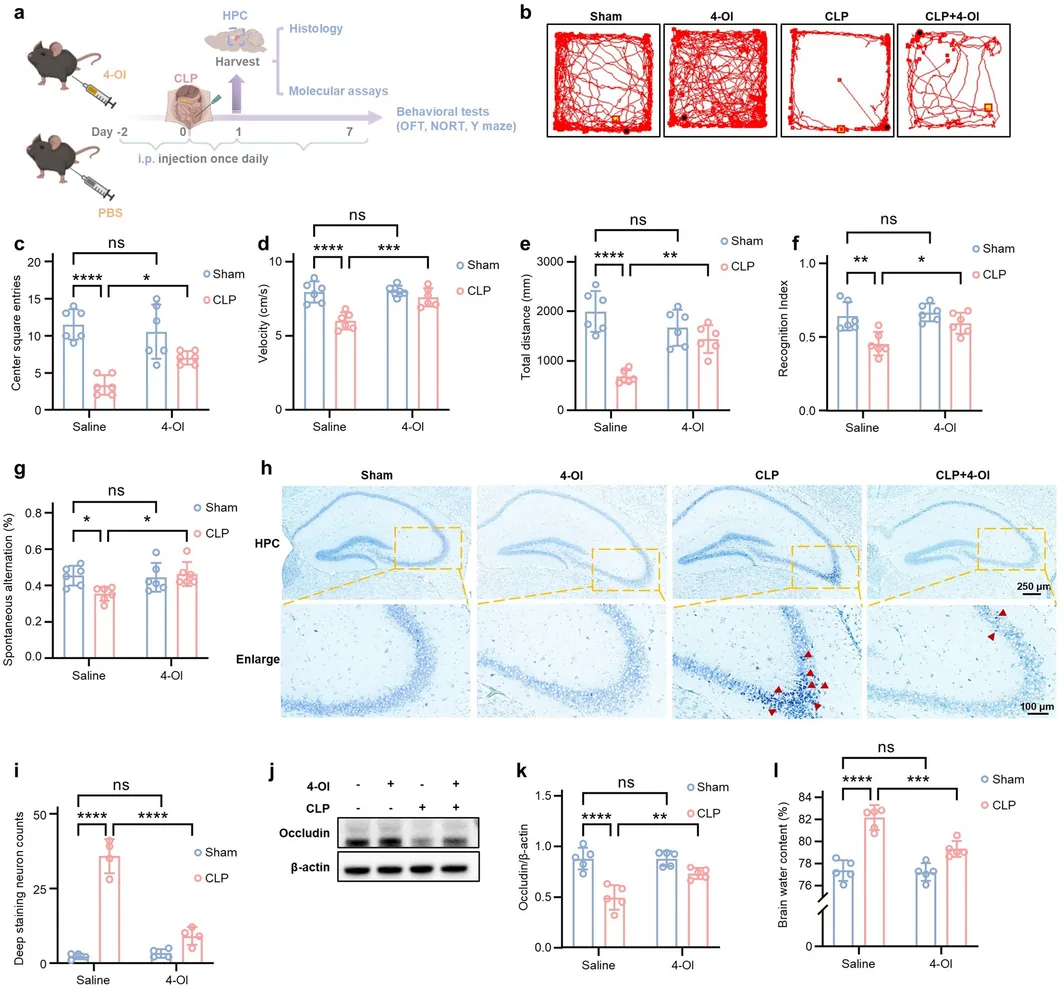
4‐OI alleviates sepsis‐associated neurobehavioral deficits and neurovascular dysfunction. (a) Experimental timeline showing 4‐OI administration, CLP surgery, hippocampal tissue collection, and behavioral testing. Mice were treated with 4‐OI or saline before CLP. Hippocampal tissues were harvested 24 h after CLP, and behavioral tests were performed from day 7 to day 9. HPC, hippocampus. (b) Representative tracking plots of mice from different experimental groups in the open field test, *n* = 6 mice per group. (c) Quantification of center zone entries (c), movement velocity (d), and total distance traveled (e) in the open field test. (f) Recognition index (RI) in the novel object recognition test among different experimental groups of mice, *n* = 6 mice per group. (g) Statistical analysis of spontaneous alternation percentage in the Y‐maze spontaneous alternation test in mice from different experimental groups, *n* = 6 mice per group. (h) Representative Nissl staining images and quantitative analysis (i) of hippocampal neurons in CLP mice pretreated with 4‐OI (50 mg/kg). Red arrows indicate dark, shrunken Nissl bodies. Scale bars, 200 μm (left) and 50 μm (right), *n* = 4 mice per group. (j) Representative Western blot images and densitometric quantification (k) of hippocampal occludin protein levels, *n* = 5 mice per group. (l) Brain water content was determined using the wet‐to‐dry weight method to evaluate cerebral edema, *n* = 5 mice per group. For animal experiments, *n* indicates the number of mice per group. Data in panels c–g, i and k, l were analyzed by two‐way ANOVA followed by Tukey's multiple comparisons test. *p < 0.05, **p < 0.01, ***p < 0.001, and ****p < 0.0001.

To further evaluate whether 4‐OI remains effective when administered after septic challenge, mice received 4‐OI or saline 2 h after CLP or sham surgery. Post‐treatment with 4‐OI reduced the number of deeply stained degenerating neurons in the hippocampus 24 h after CLP (Figure S2a,b). In parallel, 4‐OI post‐treatment improved cognitive performance, as shown by increased recognition index in the novel object recognition test and increased spontaneous alternation percentage in the Y‐maze test after CLP (Figure S2c,d). These results suggest that 4‐OI retains neuroprotective effects even when administered after CLP.

Nissl staining showed increased deeply stained degenerating neurons in the pretreatment model, whereas 4‐OI reduced these pathological changes (Figure 1g,h). CLP also decreased hippocampal occludin expression, increased brain water content, and enhanced Evans blue extravasation, indicating blood–brain barrier disruption and cerebral edema; these effects were alleviated by 4‐OI pretreatment (Figure 1i–k; Figure S1b). In addition, CLP increased hippocampal *Il6*, *Il1b*, and *Tnf* mRNA levels, which were suppressed by 4‐OI (Figure S1c). These results indicate that 4‐OI protects against CLP‐induced neurobehavioral deficits, hippocampal injury, blood–brain barrier dysfunction, and inflammation.

### 3.2 4‐OI Suppresses Hippocampal NET Formation and Promotes Microglial Apoptotic Cell Clearance

Previous studies have linked NET formation to septic neurovascular injury and cognitive dysfunction, and 4‐OI has been shown to regulate NET release [22]. We examined whether 4‐OI affects NET formation after CLP. Immunofluorescence staining showed increased CitH3/MPO colocalization in the hippocampus of CLP mice and in LPS‐stimulated BMDNs, indicating enhanced NET formation (Figure 2a–d). 4‐OI pretreatment markedly reduced these NET‐associated signals. Western blot analysis further confirmed that CLP‐induced upregulation of hippocampal MPO and CitH3 was suppressed by 4‐OI (Figure 2e–g).

**FIGURE 2:**
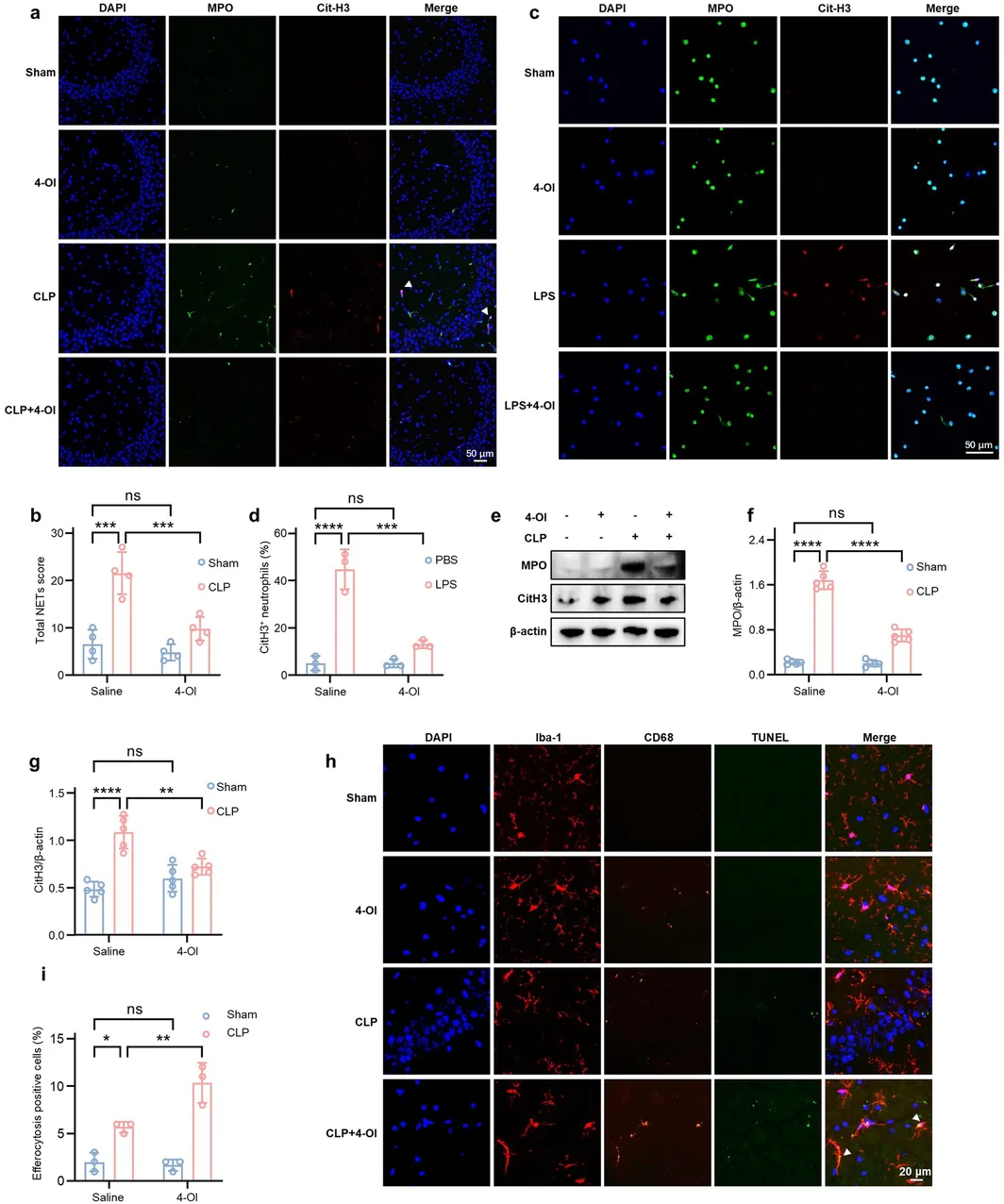
4‐OI limits NET formation and improves microglial efferocytosis after CLP. (a) Representative immunofluorescence images and quantitative analysis (b) showing co‐localization of MPO and CitH3 in the hippocampal region. White arrowheads indicate MPO/CitH3 double‐positive NET‐like structures. Scale bars, 50 μm, *n* = 4 mice per group. (c) Representative immunofluorescence images and quantitative analysis (d) showing colocalization of MPO and CitH3 in LPS‐stimulated BMDNs. Scale bars, 50 μm, *n* = 3 independent experiments. (e) Representative Western blot images and densitometric quantification (f, g) of hippocampal MPO and CitH3 protein levels, *n* = 5 mice per group. (h) Representative immunofluorescence images and quantitative analysis (i) showing colocalization of Iba‐1, CD68 and TUNEL in the hippocampal region. White arrowheads indicate Iba‐1‐positive microglia containing CD68‐positive compartments associated with TUNEL‐positive apoptotic cells, consistent with microglial efferocytosis. Scale bars, 20 μm, *n* = 3 mice per group. For animal experiments, *n* indicates the number of mice per group; for BMDNs cell experiments, *n* indicates independent cell culture experiments. Data in panels b, d, f, g and i were analyzed by two‐way ANOVA followed by Tukey's multiple comparisons test. **p < 0.01, ***p < 0.001, and ****p < 0.0001.

We then assessed microglial interaction with apoptotic cells. In the septic hippocampus, CLP mice showed limited Iba‐1/CD68/TUNEL triple‐positive structures, whereas 4‐OI pretreatment markedly increased the proportion of Iba‐1‐positive microglia associated with CD68‐positive compartments containing TUNEL‐positive apoptotic material (Figure 2h,i). These findings suggest that 4‐OI enhances microglial engagement with apoptotic cells, consistent with improved efferocytic activity during septic injury.

### 3.3 NRF2 Activation Mediates 4‐OI‐Induced Suppression of NET Formation

Previous studies have shown that 4‐OI activates the KEAP1‐NRF2 cytoprotective pathway. We therefore asked whether NRF2 signaling contributes to the inhibitory effect of 4‐OI on NET formation during septic challenge. Western blot analysis showed a modest increase in hippocampal NRF2 expression after CLP, which was further enhanced by 4‐OI pretreatment and attenuated by ML385 (Figure 3a–d). Immunofluorescence staining showed that 4‐OI reduced hippocampal MPO/CitH3 colocalization after CLP, whereas ML385 partially reversed this effect (Figure 3e,f).

**FIGURE 3:**
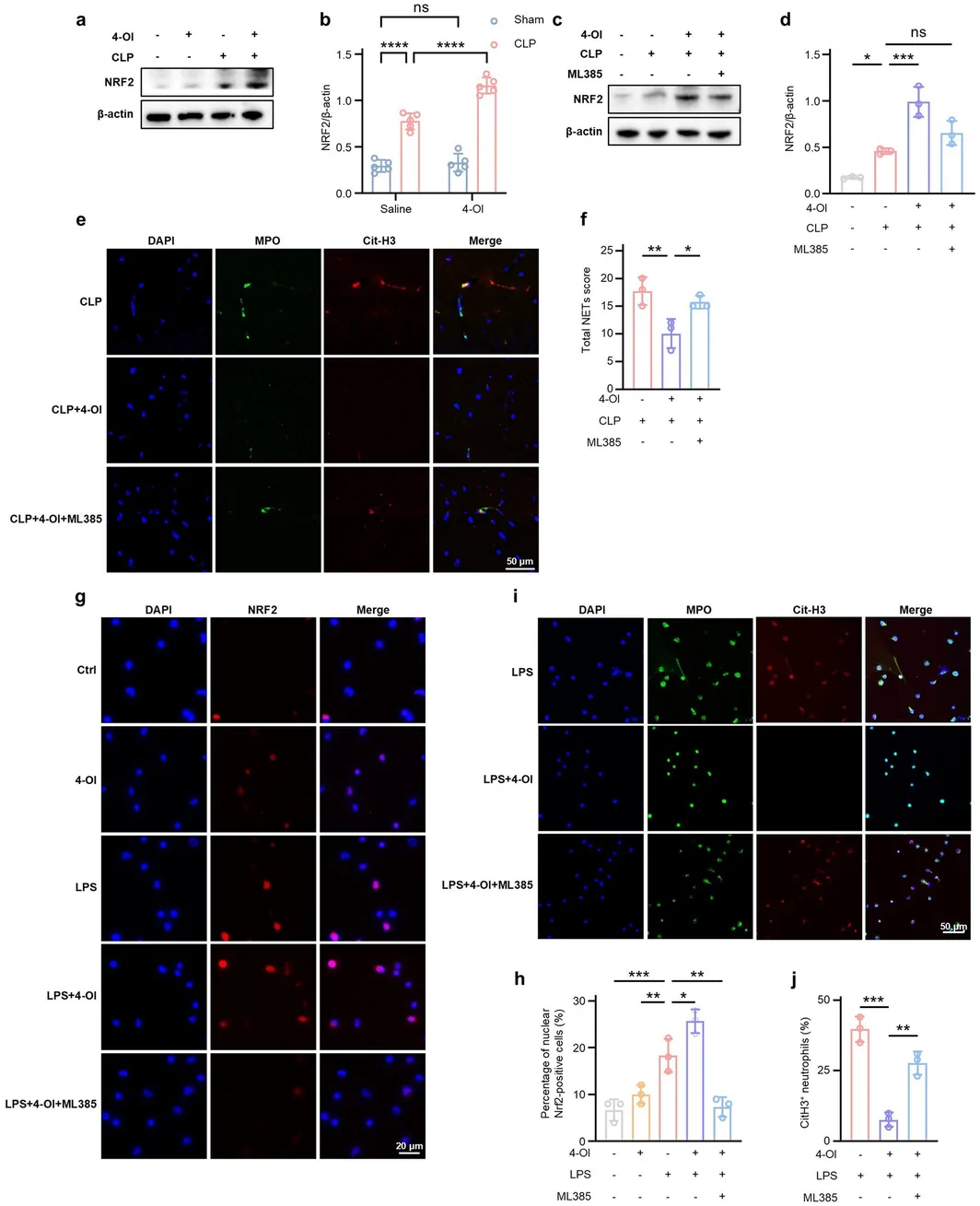
NRF2 activation contributes to 4‐OI‐mediated suppression of NET formation. (a) Representative Western blot images and densitometric quantification (b) of hippocampal NRF2 protein levels, *n* = 5 mice per group. (c) Representative Western blot images and densitometric quantification (d) of hippocampal NRF2 protein levels, *n* = 5 mice per group. (e) Representative immunofluorescence images and quantitative analysis (f) showing colocalization of CitH3 and MPO in the hippocampal region. Scale bars, 50 μm, *n* = 3 mice per group. (g) Representative immunofluorescence images and quantification (h) showing NRF2 nuclear translocation in LPS‐stimulated BMDNs. Scale bars, 20 μm, *n* = 3 independent experiments. (i) Representative immunofluorescence images and quantitative analysis (j) showing colocalization of MPO and CitH3 in LPS‐stimulated BMDNs. Scale bars, 50 μm, *n* = 3 independent experiments. For animal experiments, *n* indicates the number of mice per group; for BMDNs cell experiments, *n* indicates independent cell culture experiments. Data in panels b were analyzed by two‐way ANOVA followed by Tukey's multiple comparisons test. Data in panels d, f, h and j were analyzed by one‐way ANOVA followed by Tukey's multiple comparisons test. *p < 0.05, **p < 0.01, and ***p < 0.001; ns, not significant.

Similar results were observed in BMDNs. LPS modestly increased NRF2 expression, while 4‐OI further promoted NRF2 nuclear translocation; this effect was reduced by ML385 (Figure 3g,h). 4‐OI also suppressed LPS‐induced MPO/CitH3 colocalization and reduced the proportion of CitH3‐positive neutrophils, whereas ML385 partially restored NET formation (Figure 3i,j). These data indicate that NRF2 activation contributes to 4‐OI‐mediated suppression of NET formation.

### 3.4 4‐OI Downregulates Neutrophil PD‐L1 via NRF2 to Limit NET Formation

We therefore examined whether PD‐L1 participates in 4‐OI‐mediated NET regulation. CLP increased hippocampal PD‐L1 protein expression, whereas 4‐OI reduced PD‐L1 levels; this effect was attenuated by ML385 (Figure 4a–d). Immunofluorescence staining further showed increased Ly6G/PD‐L1 colocalization in the hippocampus after CLP. 4‐OI reduced Ly6G/PD‐L1 double‐positive signals, whereas ML385 partially restored them (Figure 4e,f).

**FIGURE 4:**
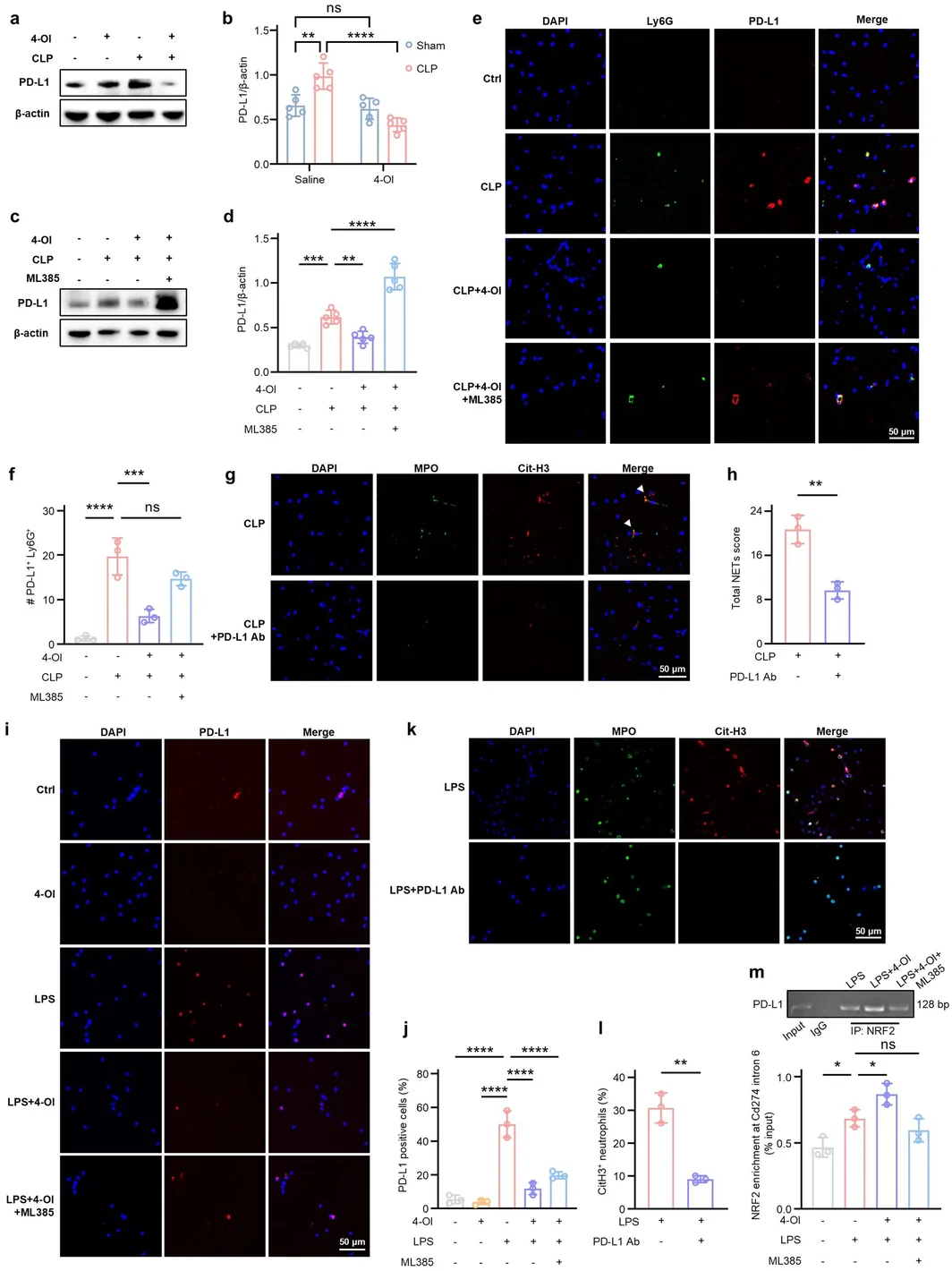
4‐OI limits PD‐L1‐mediated NET formation through NRF2 activation. (a) Representative Western blot images and densitometric quantification (b) of hippocampal PD‐L1 protein levels, *n* = 5 mice per group. (c) Representative Western blot images and densitometric quantification (d) of hippocampal PD‐L1 protein levels, *n* = 5 mice per group. (e) Representative immunofluorescence images and quantitative analysis (f) showing colocalization of Ly6G and PD‐L1 in the hippocampal region. Scale bars, 50 μm, *n* = 3 mice per group. (g) Representative immunofluorescence images and quantitative analysis (h) showing colocalization of CitH3 and MPO in the hippocampal region. Scale bars, 50 μm, *n* = 3 mice per group. (i) Representative immunofluorescence images and quantification (j) of PD‐L1 in LPS‐stimulated BMDNs. Scale bars, 50 μm, *n* = 3 independent experiments. (k) Representative immunofluorescence images and quantitative analysis (l) showing co‐localization of MPO and CitH3 in LPS‐stimulated BMDNs. Scale bars, 50 μm, *n* = 3 independent experiments. (m) Representative ChIP‐PCR bands and ChIP‐qPCR quantification showing NRF2 enrichment at the predicted *Cd274* intron 6 regulatory region in BMDNs. Input DNA and IgG were used as positive and negative controls, respectively, *n* = 3 independent experiments. For animal experiments, n indicates the number of mice per group; for BMDNs cell experiments, n indicates independent cell culture experiments. Data in panels b were analyzed by two‐way ANOVA followed by Tukey's multiple comparisons test. Data in panels d, f, h, j, l and m were analyzed by one‐way ANOVA followed by Tukey's multiple comparisons test. *p < 0.05, **p < 0.01, ***p < 0.001, and ****p < 0.0001; ns, not significant.

PD‐L1 blockade reduced MPO/CitH3 colocalization in the hippocampus of CLP mice, suggesting that PD‐L1 contributes to NET formation in vivo (Figure 4g,h). In BMDNs, 4‐OI suppressed LPS‐induced PD‐L1 expression, and ML385 partially reversed this effect (Figure 4i,j). PD‐L1 neutralization also reduced LPS‐induced MPO/CitH3 colocalization and the proportion of CitH3‐positive neutrophils (Figure 4k,l). Based on a previous report showing NRF2 enrichment at the *Cd274* intron 6 region and negative regulation of PD‐L1 in sepsis [20], we performed ChIP‐qPCR targeting the predicted *Cd274* intron 6 regulatory region. NRF2 enrichment at this region was detected after LPS stimulation and was further increased by 4‐OI treatment, whereas ML385 attenuated this enrichment (Figure 4m). These findings identify neutrophil PD‐L1 as a downstream target of the 4‐OI/NRF2 pathway involved in NET formation.

### 3.5 Transcriptomics Reveals Compensatory Activation of Microglia/Myeloid Clearance Programs in the Septic Hippocampus

To explore transcriptional programs associated with SAE, we reanalyzed two hippocampal transcriptomic cohorts, including a public SAE cohort and an independent CLP cohort generated in our laboratory. Principal component and differential expression analyses showed clear separation between control and septic hippocampal samples in both cohorts (Figure 5a; Figure S3a–c). Shared differentially expressed genes were mainly enriched in inflammatory and immune‐related pathways, including cytokine response, IL‐17 signaling, and TNF signaling (Figure 5b; Figure S3d,e).

**FIGURE 5:**
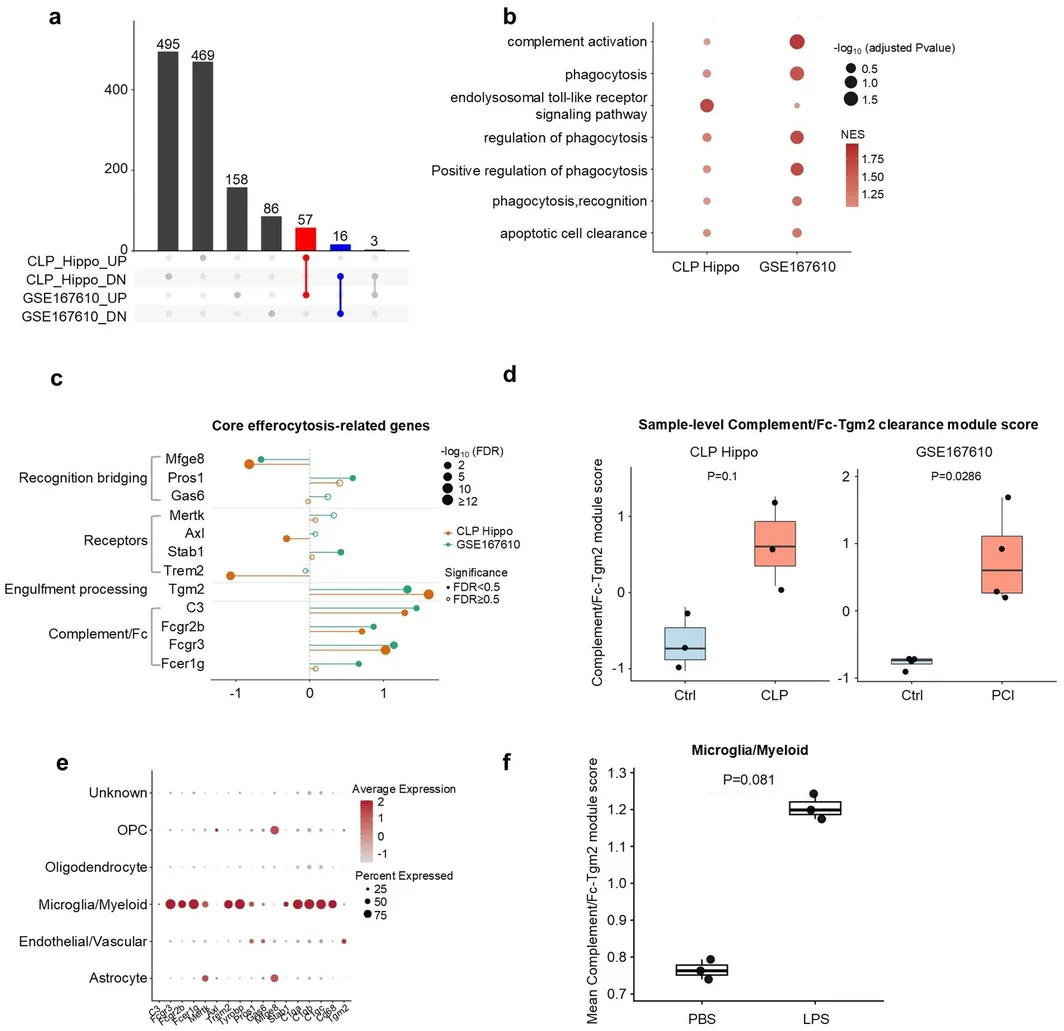
Efferocytosis‐related clearance programs are transcriptionally activated in the septic hippocampus. (a) Overlap of direction‐consistent differentially expressed genes between two hippocampal transcriptomic cohorts. (b) GSEA of efferocytosis‐related biological processes. (c) Differential expression of core efferocytosis‐related genes grouped by functional categories, including recognition bridging, receptors, engulfment processing, and complement/Fc‐associated genes. (d) Sample‐level Complement/Fc–Tgm2 module scores in bulk hippocampal RNA‐seq cohorts. (e) Expression of efferocytosis‐related genes across major brain cell types in mouse SAE scRNA‐seq data. (f) Complement/Fc–Tgm2 module scores in microglia/myeloid cells from PBS‐ and LPS‐treated mice. Dot size and color scales are indicated in each panel. The *p* values were calculated using two‐sided Wilcoxon rank‐sum tests.

Focused GSEA revealed positive enrichment of complement activation, phagocytosis, phagocytosis regulation, phagocytosis recognition, and apoptotic cell clearance pathways in septic hippocampal samples (Figure 5c; Figure S4a,b; Figure S5a). Core efferocytosis‐associated genes, including Tgm2, C3, Fcgr3, Fcgr2b, and Fcer1g, were upregulated across datasets (Figure 5d). A Complement/Fc‐Tgm2 clearance module score was also increased in septic hippocampal samples, reaching significance in the public cohort and showing a concordant upward trend in the independent CLP cohort (Figure 5e).

Single‐cell RNA‐seq analysis showed that the Complement/Fc–Tgm2 clearance module was predominantly enriched in microglia/myeloid cells (Figure 5f,g; Figure S6a–c). Thus, efferocytosis‐related clearance programs are transcriptionally activated in septic hippocampus and mainly associated with the microglia/myeloid compartment. Importantly, this transcriptional activation should not be interpreted as evidence of enhanced efferocytic function. Instead, it may reflect a compensatory response of microglia/myeloid cells to increased apoptotic burden and inflammatory injury after CLP. Consistent with this interpretation, our in vivo staining showed impaired efferocytosis‐associated clearance in the septic hippocampus despite upregulation of clearance‐related genes (see Figure 2h,i). We therefore hypothesized that septic microenvironmental factors may limit the execution of this transcriptionally activated clearance program. Given the prominent accumulation of NETs in the septic hippocampus, we next examined whether NETs directly impair microglial efferocytic function.

### 3.6 NETs Directly Impair Microglial Efferocytosis

Based on the transcriptomic enrichment of efferocytosis‐related programs and the accumulation of NETs in the septic hippocampus, we next examined whether NETs contribute to defective microglial clearance of apoptotic cells. Primary mouse microglia were isolated and incubated with CFSE‐labeled apoptotic HT22 cells in the presence or absence of isolated NETs (Figure 6a). Immunofluorescence staining showed that NET exposure reduced the uptake of CFSE‐labeled apoptotic HT22 cells by Iba‐1‐positive primary microglia, whereas DNase I‐mediated NET degradation restored apoptotic cell engulfment (Figure 6b,c). Consistently, flow cytometry demonstrated a reduced proportion of CFSE‐positive Iba‐1‐positive primary microglia after NET treatment, which was reversed by DNase I pretreatment (Figure 6d,e).

**FIGURE 6:**
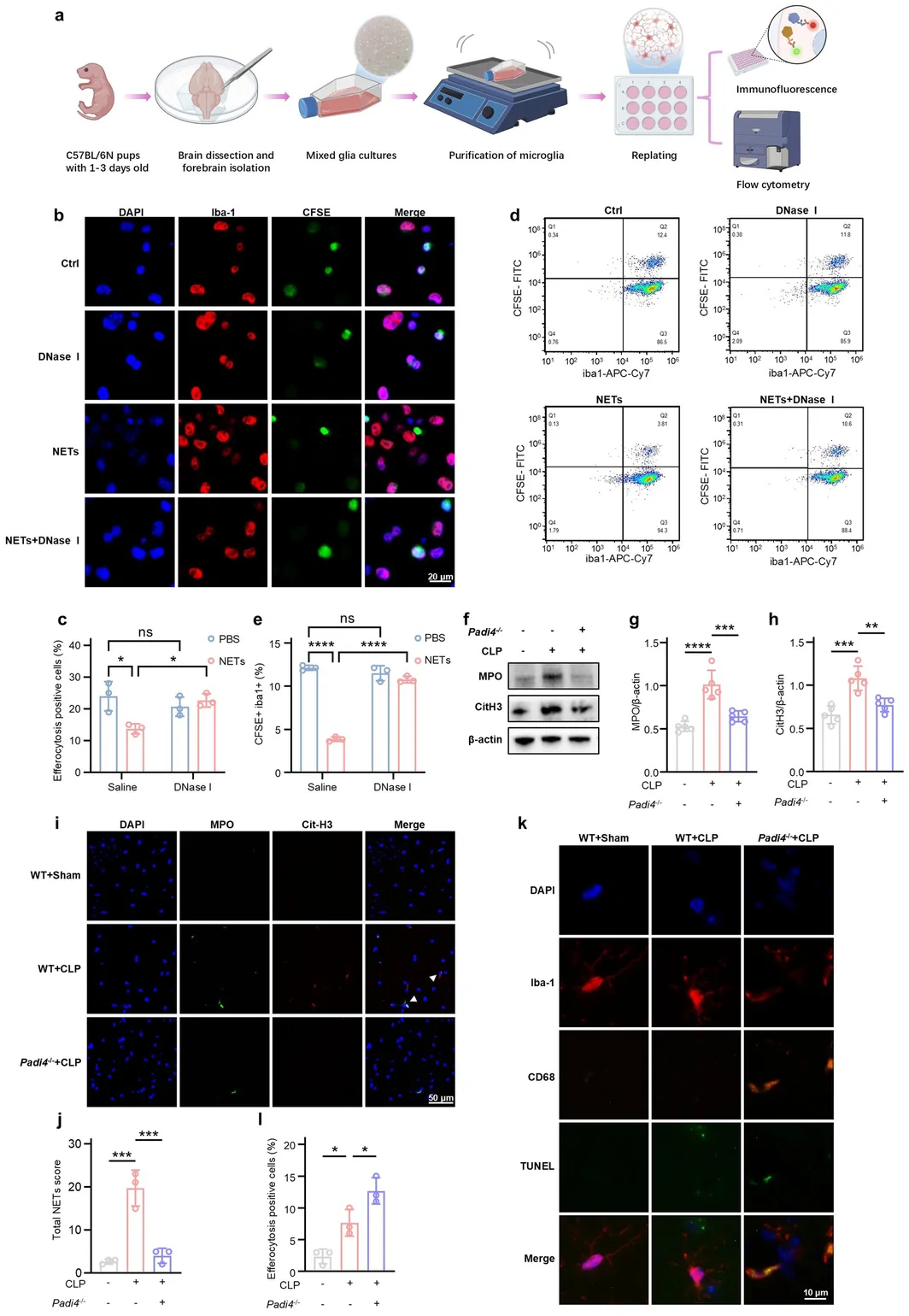
NET degradation or PAD4 deficiency restores microglial efferocytosis during septic challenge. (a) Workflow for primary microglia isolation and analysis. Forebrains from C57BL/6N pups aged 1–3 days were used to establish mixed glial cultures. Purified microglia were replated for immunofluorescence staining and flow cytometry. (b) Representative immunofluorescence images and quantification (c) of efferocytosis of CFSE‐labeled apoptotic HT22 cells by primary microglia. Scale bars, 50 μm, *n* = 3 independent experiments. (d) Representative flow cytometry plots and quantification (e) of CFSE‐positive Iba‐1‐positive primary microglia, indicating microglia that engulfed CFSE‐labeled apoptotic HT22 cells, *n* = 3 independent experiments. (f) Representative Western blot images and densitometric quantification (g, h) of hippocampal MPO and CitH3 protein levels, *n* = 5 mice per group. (i) Representative immunofluorescence images and quantitative analysis (j) showing colocalization of CitH3 and MPO in the hippocampal region. Scale bars, 50 μm, *n* = 3 mice per group. (k) Representative immunofluorescence images and quantitative analysis (l) showing colocalization of Iba‐1, CD68 and TUNEL in the hippocampal region. Scale bars, 25 μm, *n* = 3 mice per group. For animal experiments, *n* indicates the number of mice per group; for primary microglia experiments, *n* indicates independent cell culture experiments. Data in panels c and e were analyzed by two‐way ANOVA followed by Tukey's multiple comparisons test. Data in panels g, h, j and l were analyzed by one‐way ANOVA followed by Tukey's multiple comparisons test. *p < 0.05, **p < 0.01, ***p < 0.001, and ****p < 0.0001.

To further support this finding, we also examined BV2 microglia‐like cells incubated with CFSE‐labeled apoptotic neutrophils. Similarly, NET exposure reduced the engulfment of apoptotic neutrophils by BV2 cells, whereas DNase I treatment restored this efferocytic activity (Figure S7a–d). Together, these results support a direct inhibitory effect of NETs on microglial efferocytosis in vitro.

To assess the role of NETs in vivo, *Padi4* ^*−/−*^ mice were subjected to CLP. CLP increased hippocampal MPO and CitH3 protein expression and MPO/CitH3 colocalization in WT mice, whereas these changes were attenuated in *Padi4* ^*−/−*^ mice (Figure 6f–j). Notably, compared with WT CLP mice, *Padi4* ^−/−^ CLP mice showed increased Iba‐1/CD68/TUNEL triple‐positive structures in the hippocampus, indicating enhanced association of apoptotic material with CD68‐positive microglial phagolysosomal compartments (Figure 6k,l). Together with the in vitro findings, these results suggest that NET accumulation contributes to impaired microglial efferocytosis during septic brain injury.

## 4 Discussion

Our study identifies a NET‐centered neuroinflammatory mechanism that contributes to SAE progression. We found that septic challenge induced blood–brain barrier disruption, hippocampal NET accumulation, impaired microglial efferocytosis, and cognitive dysfunction. Functional experiments further showed that NETs directly impaired the efferocytic activity of microglia, whereas DNase I‐mediated NET degradation restored apoptotic cell clearance in vitro. PAD4 deficiency reduced hippocampal NET accumulation and enhanced microglial association with apoptotic cells in vivo. These findings suggest that excessive NET formation may serve as a critical link between neutrophil activation and defective microglial clearance in the septic brain. Importantly, 4‐OI markedly attenuated NET formation and improved neurological outcomes in septic mice. Mechanistically, 4‐OI activated NRF2 signaling in neutrophils and suppressed PD‐L1 expression, thereby limiting NET release. Together, our findings identify a 4‐OI‐regulated NRF2/PD‐L1/NETs pathway that attenuates neuroinflammation by preserving microglial efferocytosis in SAE.

Previous studies have shown that sepsis induces systemic NET formation in endotoxemia and CLP models and that circulating cfDNA and NET‐associated biomarkers correlate with disease severity and organ dysfunction in septic patients [22, 28]. PAD4‐mediated histone citrullination facilitates chromatin decondensation and NET formation, while PAD4 deficiency reduces NET generation and attenuates sepsis‐related organ injury [29, 30]. Consistent with these findings, we detected increased MPO/CitH3 colocalization and elevated MPO and CitH3 protein levels in the hippocampus after CLP. These changes were accompanied by reduced microglial clearance of apoptotic cells. Given the susceptibility of the hippocampus to inflammatory and vascular disturbances, local NET accumulation may promote sustained neuroinflammation and contribute to hippocampus‐dependent cognitive dysfunction after sepsis. These findings provide further rationale for targeting pathological NET accumulation in septic brain injury.

4‐OI has been shown to restrain NET formation in neutrophils exposed to inflammatory stimuli, including LPS, suggesting that 4‐OI modulates neutrophil effector responses in addition to its established anti‐inflammatory effects [18]. Consistent with this, we found that 4‐OI markedly reduced NET‐associated MPO/CitH3 colocalization and decreased MPO and CitH3 protein levels in the hippocampi of mice subjected to CLP. In vitro, 4‐OI attenuated LPS‐induced NET formation in bone marrow‐derived neutrophils. Together, these findings identify the suppression of NET formation as an important component of the neuroprotective effects of 4‐OI during septic brain injury.

A recent study by Wu et al. reported that 4‐OI alleviates SAE by inhibiting small intestinal γδ T17 cell migration and reducing STING/C1q‐mediated microglial synaptic pruning, supporting the protective potential of 4‐OI in SAE [31]. Distinct from this gut–meningeal–microglial immune axis, our study identifies a neutrophil‐centered NRF2/PD‐L1/NETs pathway through which 4‐OI preserves microglial efferocytosis and limits septic neuroinflammation. Thus, our findings extend the current understanding of 4‐OI‐mediated neuroprotection by linking neutrophil NET formation to defective microglial clearance in SAE.

NRF2 is an important regulator of neutrophil redox homeostasis, and its activation has been reported to limit ROS‐dependent NET formation and reduce sepsis‐induced injury [32]. Increased PD‐L1 expression on neutrophils and monocytes has been associated with immune dysfunction and adverse outcomes in sepsis [24, 25, 26, 27]. Moreover, neutrophil PD‐L1 has been shown to promote GSDMD‐dependent NET release in experimental SAE [12]. In our study, 4‐OI enhanced NRF2 signaling and reduced PD‐L1 expression and NET formation in vivo and in LPS‐stimulated BMDNs. These effects were weakened by ML385, while PD‐L1 neutralization suppressed NET formation. ChIP‐qPCR further showed increased NRF2 enrichment at the *Cd274* intron 6 region after 4‐OI treatment, which was attenuated by ML385, supporting a potential NRF2‐mediated regulation of PD‐L1, although indirect mechanisms may also contribute.

Microglial efferocytosis is essential for clearing apoptotic cells in the CNS and preventing secondary necrosis and persistent inflammation [3, 4]. Recent evidence further suggests that enhancing microglial efferocytosis can alleviate neuroinflammation in SAE [33]. Transcriptomic analysis showed upregulated efferocytosis‐related clearance programs in the septic hippocampus, particularly in microglia/myeloid cells, likely reflecting a compensatory response to apoptotic and inflammatory stress rather than enhanced efferocytosis. Consistently, septic hippocampi showed insufficient microglial engagement with apoptotic cells, and NETs directly impaired primary microglial efferocytosis in vitro. These findings are consistent with previous reports that NETs suppress macrophage efferocytosis and perpetuate inflammatory responses [34, 35]. Importantly, DNase I‐mediated NET degradation restored microglial efferocytic activity, whereas PAD4 deficiency reduced hippocampal NET formation and enhanced Iba‐1/CD68/TUNEL triple‐positive structures after CLP. Together, these results support NET accumulation as a functional contributor to impaired microglial efferocytosis during septic neuroinflammation. Although the underlying mechanisms remain unresolved, studies in macrophages suggest that persistent NETs can impair efferocytosis by promoting proteolytic cleavage of MerTK [14]. In addition, NET‐derived DNA, histones, and proteases may activate inflammatory signaling pathways in microglia. Whether these mechanisms mediate defective microglial efferocytosis in SAE requires further investigation. In addition, we also found that 4‐OI administered 2 h after CLP reduced hippocampal neuronal injury and improved cognitive performance, suggesting that 4‐OI retains protective effects after septic challenge. However, the NRF2/PD‐L1/NETs mechanism was mainly validated under the pretreatment paradigm, and whether delayed 4‐OI acts through the same pathway requires further investigation.

Taken together, our study identifies NET accumulation as a mechanistic link between neutrophil activation and impaired microglial efferocytosis in SAE. Mechanistically, 4‐OI activates NRF2, suppresses neutrophil PD‐L1 expression, and consequently limits NET formation, thereby restoring microglial efferocytic function.

## 5 Conclusions

In conclusion, our study demonstrates that excessive NET formation contributes to neuroinflammation and cognitive dysfunction in SAE. By activating NRF2 and suppressing neutrophil PD‐L1 expression, 4‐OI limits NET formation, preserves microglial efferocytosis, and alleviates septic brain injury. These findings suggest that targeting the NRF2/PD‐L1/NETs axis may provide a potential therapeutic strategy for SAE.

## Author Contributions

Weidong Gu and Lina Huang were responsible for conceptualization, study design and manuscript editing. Na Meng and Xixue Zhang contributed to the experiments, data analysis, visualization and manuscript composition. Kaiwen Tian, Yue Zhou, Lei Gao, Fafa Sun and Yulin Zhang contributed to data curation. All authors reviewed and approved the final version of the manuscript.

## Funding

This work was supported by the National Natural Science Foundation of China, 82271286, 82302417. Huadong Hospital Key Discipline Project, ZDXK2210. Seed Program for Medical New Technology Research and Transformation of Shanghai Municipal Health Commission, 2024ZZ2031.

## Ethics Statement

Animal studies were conducted under protocols approved by the Animal Use Committee of Shanghai General Hospital, Shanghai Jiao Tong University (No. 2023AW062).

## Conflicts of Interest

The authors declare no conflicts of interest.

## Supporting information


**Figure S1:** 4‐OI alleviates CLP‐induced blood–brain barrier disruption and hippocampal inflammation.
(a) Kaplan–Meier survival analysis after CLP. Survival was monitored for 72 h after sham or CLP surgery. Survival curves were compared using the log‐rank (Mantel–Cox) test (*χ* ^2^ = 4.423, df = 2, *p* = 0.1095). (b) Representative images of Evans blue dye extravasation in brain tissues and quantitative analysis of Evans blue dye content, expressed as μg per *g* of brain tissue, *n* = 3 mice per group. (c) qRT‐PCR analysis showing the mRNA expression levels of *Il1b*, *Il6* and *Tnf* in hippocampal tissues, *n* = 3 mice per group. For animal experiments, *n* indicates the number of mice per group. Data in panels b and c were analyzed by two‐way ANOVA followed by Tukey's multiple comparisons test. *p < 0.05, **p < 0.01, ***p < 0.001, and ****p < 0.0001.
**Figure S2:** Post‐treatment administration of 4‐OI alleviates hippocampal neuronal injury and cognitive impairment after CLP. (a) Representative Nissl staining images and quantitative analysis (b) of hippocampal neurons in CLP mice treated with 4‐OI (50 mg/kg). Red arrows indicate dark, shrunken Nissl bodies. Scale bars, 200 μm (left) and 50 μm (right), *n* = 4 mice per group. (c) Recognition index (RI) in the novel object recognition test among different experimental groups of mice, *n* = 6 mice per group. (d) Statistical analysis of spontaneous alternation percentage in the Y‐maze spontaneous alternation test in mice from different experimental groups, *n* = 6 mice per group. For animal experiments, *n* indicates the number of mice per group. Data in panels b–d were analyzed by two‐way ANOVA followed by Tukey's multiple comparisons test. *p < 0.05, ***p < 0.001, and ****p < 0.0001.
**Figure S3:** Cross‐cohort transcriptomic analysis reveals conserved inflammatory signatures in the septic hippocampus. (a) PCA of hippocampal transcriptomic profiles from the independent CLP RNA‐seq cohort and GSE167610. (b) Volcano plots showing differentially expressed genes. (c) Log2FC concordance analysis between the two cohorts. Direction‐consistent upregulated and downregulated genes are shown in red and blue, respectively. (d) GO biological process and (e) KEGG pathway enrichment analyses of direction‐consistent seed genes. Dot size represents gene count, and color indicates –log10 (adjusted *p* value).
**Figure S4:** Representative GSEA enrichment curves of phagocytosis‐ and complement‐related pathways. (a) GSEA enrichment plots showing positive enrichment of the phagocytosis pathway in the independent CLP hippocampal cohort and GSE167610. (b) GSEA enrichment plots showing positive enrichment of the complement activation pathway in the two hippocampal transcriptomic cohorts. NES, nominal *p* value, and FDR are shown in each panel.
**Figure S5:** Pathway–gene network of efferocytosis‐related clearance programs. (a) Network visualization showing the relationships between enriched efferocytosis‐related pathways and associated core genes. Blue square nodes represent enriched biological processes, red circular nodes indicate core efferocytosis‐associated genes, and connecting lines denote gene‐pathway associations.
**Figure S6:** Single‐cell mapping of the Complement/Fc‐Tgm2 clearance module in SAE brain tissue. (a) UMAP visualization of major brain cell populations in the public mouse SAE single‐cell RNA‐seq cohort. (b) UMAP projection of the Complement/Fc‐Tgm2 clearance module score. (c) Violin plot showing the distribution of Complement/Fc‐Tgm2 module scores across major cell types. The module score was highest in the microglia/myeloid compartment.
**Figure S7:** NETs impair BV2 efferocytosis of apoptotic neutrophils, which is restored by DNase I pretreatment. (a) Representative immunofluorescence images and quantification (b) of efferocytosis of CFSE‐labeled apoptotic neutrophils by BV2 cells. Scale bars, 50 μm, *n* = 3 independent experiments. (c) Representative flow cytometry plots and quantification (d) of CFSE‐positive Iba‐1‐positive BV2 cells, indicating microglia that engulfed CFSE‐labeled apoptotic neutrophils, *n* = 3 independent experiments. For BV2 cell experiments, *n* indicates independent cell culture experiments. Data in panels b and d were analyzed by two‐way ANOVA followed by Tukey's multiple comparisons test. *p < 0.05, **p < 0.01, and ****p < 0.0001.

**Supplementary File 1** Statistical information. Detailed statistical information for the analyses presented in the main and supplementary figures, including the statistical tests used, test statistics, degrees of freedom, exact *p* values, and post hoc multiple‐comparison results.

**Supplementary File 2** RNA sequencing. Processed gene expression data and differential‐expression results from the in‐house CLP hippocampal RNA‐sequencing dataset used in the present study.

**Table S1:** Details of antibodies.
**Table S2:** List of qPCR primers.
**Table S3:** Primers for ChIP‐qPCR.

## Data Availability

The hippocampal RNA‐sequencing dataset analyzed in this study was previously generated and the raw sequencing data were provided as supplementary files in our published work [27]. The processed expression matrix and differential‐expression results used in the present study are provided as Supplementary File 2. The data that support the findings of this study are available from the corresponding author upon reasonable request.
