# Supplementary material for: 4‐Octyl Itaconate Restores Microglial Efferocytosis by Suppressing NRF2/PD‐L1‐Dependent Neutrophil NET Formation in Sepsis‐Associated Encephalopathy
Source: CNS Neurosci Ther. 2026 Nov 10;32(11):e71268. doi: 10.1002/cns.71268 (PMC13752472; doi:10.1002/cns.71268)
Supplement: Supplementary file 1 [file CNS-32-e71268-s002.docx]

**Figure S1 4-OI alleviates CLP-induced blood-brain barrier disruption and hippocampal inflammation.**

a) Kaplan–Meier survival analysis after CLP. Survival was monitored for 72 h after sham or CLP surgery. Survival curves were compared using the log-rank Mantel–Cox test (χ² = 4.423, df = 2, P = 0.1095).
b) Representative images of Evans blue dye extravasation in brain tissues and quantitative analysis of Evans blue dye content, expressed as μg per g of brain tissue, n = 3 mice per group.
c) qRT-PCR analysis showing the mRNA expression levels of *Il1b*, *Il6* and *Tnf* in hippocampal tissues, n = 3 mice per group. For animal experiments, n indicates the number of mice per group. Data in panels b and c were analyzed by two-way ANOVA followed by Tukey’s multiple comparisons test. *p < 0.05, **p < 0.01, ***p < 0.001, and ****p < 0.0001.

**Figure S2 Post-treatment administration of 4-OI alleviates hippocampal neuronal injury and cognitive impairment after CLP.**

a) Representative Nissl staining images and quantitative analysis (b) of hippocampal neurons in CLP mice treated with 4-OI (50 mg/kg). Red arrows indicate dark, shrunken Nissl bodies. Scale bars, 200 μm (left) and 50 μm (right), n = 4 mice per group.
c) Recognition index (RI) in the novel object recognition test among different experimental groups of mice, n = 6 mice per group.
d) Statistical analysis of spontaneous alternation percentage in the Y-maze spontaneous alternation test in mice from different experimental groups, n = 6 mice per group. For animal experiments, n indicates the number of mice per group. Data in panels b-d were analyzed by two-way ANOVA followed by Tukey’s multiple comparisons test. *p < 0.05, ***p < 0.001, and ****p < 0.0001.

**Figure S3 Cross-cohort transcriptomic analysis reveals conserved inflammatory signatures in the septic hippocampus.**

a) PCA of hippocampal transcriptomic profiles from the independent CLP RNA-seq cohort and GSE167610.
b) Volcano plots showing differentially expressed genes.
c) Log2FC concordance analysis between the two cohorts. Direction-consistent upregulated and downregulated genes are shown in red and blue, respectively.
d) GO biological process and KEGG pathway (e) enrichment analyses of direction-consistent seed genes. Dot size represents gene count, and color indicates -log10 (adjusted P value).

**Figure S4 Representative GSEA enrichment curves of phagocytosis- and complement-related pathways.**

a) GSEA enrichment plots showing positive enrichment of the phagocytosis pathway in the independent CLP hippocampal cohort and GSE167610.
b) GSEA enrichment plots showing positive enrichment of the complement activation pathway in the two hippocampal transcriptomic cohorts. NES, nominal P value, and FDR are shown in each panel.

**Figure S5 Pathway-gene network of efferocytosis-related clearance programs.**

a) Network visualization showing the relationships between enriched efferocytosis-related pathways and associated core genes. Blue square nodes represent enriched biological processes, red circular nodes indicate core efferocytosis-associated genes, and connecting lines denote gene-pathway associations.

**Figure S6 Single-cell mapping of the Complement/Fc-Tgm2 clearance module in SAE brain tissue.**

a) UMAP visualization of major brain cell populations in the public mouse SAE single-cell RNA-seq cohort.

b) UMAP projection of the Complement/Fc-Tgm2 clearance module score.

c) Violin plot showing the distribution of Complement/Fc-Tgm2 module scores across major cell types. The module score was highest in the microglia/myeloid compartment.

**Figure S7 NETs impair BV2 efferocytosis of apoptotic neutrophils, which is restored by DNase I pretreatment.**

a)	Representative immunofluorescence images and quantification (b) of efferocytosis of CFSE-labeled apoptotic neutrophils by BV2 cells. Scale bars, 50 μm, n = 3 independent experiments.

c) Representative flow cytometry plots and quantification (d) of CFSE-positive Iba-1-positive BV2 cells, indicating microglia that engulfed CFSE-labeled apoptotic neutrophils, n = 3 independent experiments. For BV2 cell experiments, n indicates independent cell culture experiments. Data in panels b and d were analyzed by two-way ANOVA followed by Tukey’s multiple comparisons test. *p < 0.05, **p < 0.01, and ****p < 0.0001.
