# Supplementary material for: 4‐Octyl Itaconate Restores Microglial Efferocytosis by Suppressing NRF2/PD‐L1‐Dependent Neutrophil NET Formation in Sepsis‐Associated Encephalopathy
Source: CNS Neurosci Ther. 2026 Nov 10;32(11):e71268. doi: 10.1002/cns.71268 (PMC13752472; doi:10.1002/cns.71268)
Supplement: Supplementary file 2 [file CNS-32-e71268-s003.docx]

**Table S1: Details of Antibodies**

| **Antibodies** | **SOURCE** | **IDENTIFIER** |
| --- | --- | --- |
| Anti-NRF2 | Abclonal | Cat# A0674 |
| Anti-Iba-1 | Abcam | Cat# ab178846 |
| Anti-PD-L1 | Abcam | Cat# ab213480 |
| Anti-MPO | Proteintech | Cat No. 81610-1-RR |
| Histone H3 citrulline R2/R8/R17 | Abcam | Cat# ab281584 |
| Anti-Occludin | Abcam | Cat# ab216327 |
| Anti-Ly6G | Proteintech | Cat No. 32346-1-AP |
| Anti-CD68 | Biolegend | Cat# 137003 |
| anti-β-actin | Servicebio | GB11001-100 |

**Table S2: List of qPCR primers**

| **Gene** | **Forward** | **Reverse** |
| --- | --- | --- |
| Mouse-*Il6* | CCGGAGAGGAGACTTCACAG | CAGAATTGCCATTGCACAAC |
| Mouse-*Il1b* | GGATGAGGACATGAGCACCT | AGCTCATATGGGTCCGACAG |
| Mouse-*Tnf* | CCCCAAAGGGATGAGAAGTT | CACTTGGTGGTTTGCTACGA |
| Mouse-*Gapdh* | ACAGCAGTGTGCAGTTGATGA | GGCAGTCATGTCCGGTGATG |

**Table S3: Primers for ChIP-qPCR**

| **Primer name** | **Sequences (5’-3’)** |
| --- | --- |
| Mouse-*Cd274*-Forward① | ACAGTAAGCCCAGGCAGAAC |
| Mouse-*Cd274*-Reverse① | TCAGTCTTGAGCAAGCCAGG |
| Mouse-*Cd274*-Forward② | GTGGTTTCGCTAACTTGGGC |
| Mouse-*Cd274*-Reverse② | TCAGTCTTGAGCAAGCCAGG |
| Mouse-*Cd274*-Forward③ | ACAGTAAGCCCAGGCAGAAC |
| Mouse-*Cd274*-Reverse③ | GTTCCTCAGGGTGACTCAGC |
